# Supplementary material for: Activation of Oligonucleotide Polyanions Using Collisions, Electrons and Photons in a timsOmni Platform
Source: J Am Soc Mass Spectrom. 2026 Apr 30;37(6):1342–52. doi: 10.1021/jasms.5c00386 (PMC13237767; doi:10.1021/jasms.5c00386)
Supplement: Supplementary file 1 [file js5c00386_si_001.pdf]

Activation of oligonucleotide polyanions using collisions, electrons and photons in a timsOmni platform

Frédéric Rosu,[1] Rim Chiba,[1] Arjun Mani Mallika,[1] Athanasios Smyrnakis,[2] Jean-François Greisch,[3] Dimitris Papanastasiou,[2] Valérie Gabelica[1*]

[1] School of Pharmaceutical Sciences, University of Geneva, 1205 Geneva, Switzerland.

[2] Fasmatech Science & Technology, 15232 Chalandri, Athens, Greece.

[3] Bruker Switzerland AG, 8117 Fällanden, Switzerland.

[*] Corresponding author: valerie.gabelica@unige.ch

SUPPORTING INFORMATION

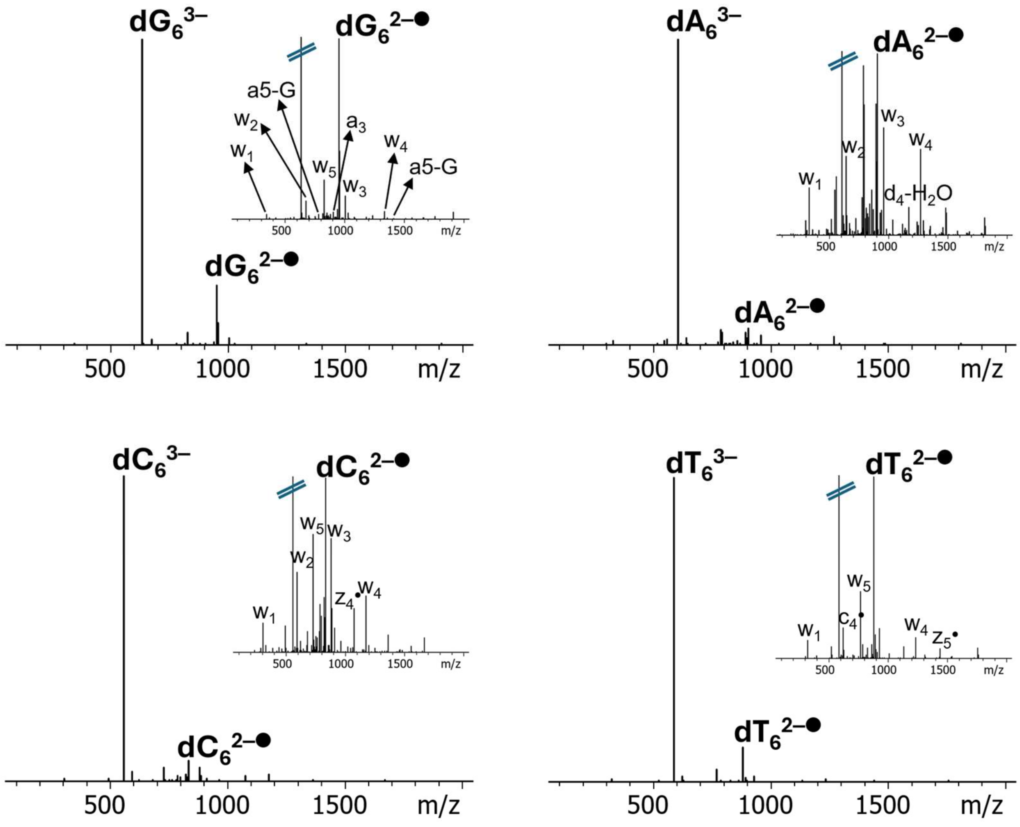

Figure S1: Product ion spectra resulting from EDD on DNA homo-hexamers dG_6^{3-}, dA_6^{3-}, dC_6^{3-} and dT_6^{3-} at 100 ms irradiation time.

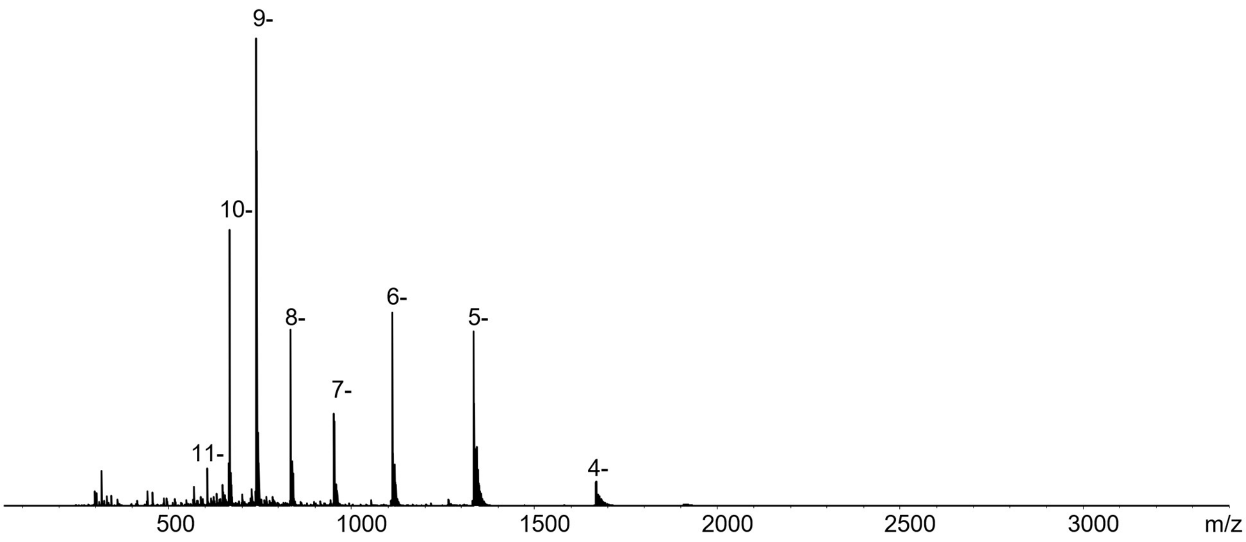

Figure S2: Representative full scan MS spectrum of Fomivirsen sprayed from 1 mM aqueous NH_4OAc and 20% MeOH.

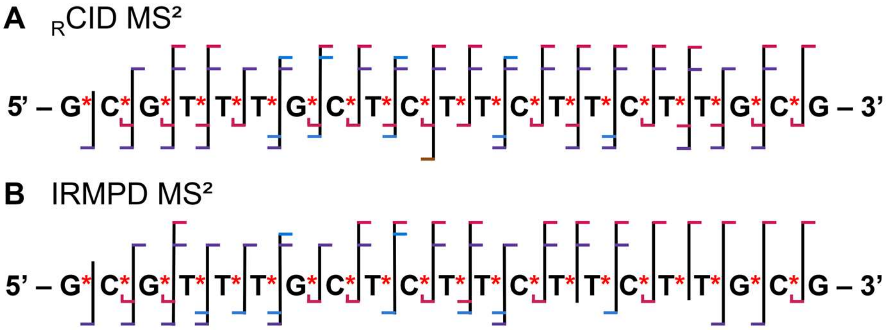

Figure S3: sequence coverage map of (**A**) $_R$CID MS² and (**B**) IRMPD MS² of Fomivirsen, $[M - 9H]^{9-}$, annotated using the nomenclature shown in Figure 1. $_R$CID amplitude and IR irradiation time were optimized to achieve informative fragmentation patterns while preserving some precursor ion intensity (on the order of ~≥50%). See Tables S5 and S6 for peak assignment with ppm errors. Only a subset of terminal fragment ions with high S/N are marked in the sequence coverage map. All other high/low intensity fragment ions of other charge states conforming the same positions are attached in supplementary Table S7. The asterisk represents Phosphorothioate backbone linkage.

Table S1: Peak list table on the fragments annotated on the sequence coverage map for Fomivirsen and that suffice to provide full sequence coverage, from the **EDD MS²** spectrum on $[M - 9H]^{9-}$. Full FAST MS output of fragment ion peaks of Fomivrisen $[M - 9H]^{9-}$ is provided in supplementary Table S7 (separate Excel file).

nt	Position (5')	Peak assignment	Charge state (z)	Observed m/z	Theoretical m/z	Mass error (ppm)	nt	Position (3')	Peak assignment	Charge state (z)	Observed m/z	Theoretical m/z	Mass error (ppm)
**G**	1	c1	1-	343.0139	343.0145	-1.75	**G**	21					
**C**	2	a2	1-	553.1038	553.1024	2.53	**C**	20	w20	7-	917.3538	917.3502	3.92
**G**	3	a3	1-	898.132	898.1321	-0.11	**G**	19	w19$^{\bullet}$	7-	873.6311	873.6314	-0.34
**T**	4	a4$^{\bullet}$	2-	608.072	608.0701	3.12	**T**	18					
**T**	5	a5$^{\bullet}$	2-	768.084	768.0817	2.99	**T**	17	w17	6-	908.7349	908.7303	5.06
**T**	6	a6$^{\bullet}$	2-	928.0959	928.0933	2.80	**T**	16	w16	6-	855.3962	855.3931	3.62
**G**	7	a7$^{\bullet}$	3-	733.4066	733.403	4.91	**G**	15	w15	6-	802.0596	802.0559	4.61
**C**	8	a8$^{\bullet}$	2-	1253.1228	1253.1199	2.31	**C**	14	w14	5-	893.6653	893.6627	2.90
**T**	9	d9	3-	980.0766	980.0725	4.18	**T**	13	z13	4-	1012.5888	1012.5858	2.96
**C**	10	a10$^{\bullet}$	3-	1043.4314	1043.4264	4.79	**C**	12	w12	5-	768.6559	768.6533	3.38
**T**	11	a11	4-	862.5785	862.5757	3.25	**T**	11	z11	4-	856.328	856.3241	4.55
**T**	12	a12	4-	942.5843	942.5815	2.97	**T**	10	w10	4-	804.8104	804.8068	4.47
**C**	13	a13$^{\bullet}$	4-	1018.591	1018.5854	5.50	**C**	9	z9	3-	928.7554	928.7524	3.23
**T**	14	a14	5-	878.8761	878.8731	3.41	**T**	8	w8	4-	648.5483	648.5451	4.93
**T**	15	a15$^{\bullet}$	6-	785.3981	785.3956	3.18	**T**	7	w7	3-	758.3904	758.3882	2.90
**C**	16	a16	5-	1003.6876	1003.6809	6.68	**C**	6	z6	2-	921.093	921.0973	-4.66
**T**	17	a17	6-	889.7414	889.738	3.82	**T**	5	w5	3-	550.0416	550.0393	4.18
**T**	18	a18$^{\bullet}$	7-	808.2105	808.2063	5.20	**T**	4	w4$^{\bullet}$	1-	665.0487	665.0471	2.41
**G**	19	a19	7-	857.5008	857.4962	5.36	**G**	3	w3	1-	1012.0884	1012.0861	2.27
**C**	20	a20	7-	901.075	901.071	4.44	**C**	2	w2	1-	667.0579	667.0564	2.25
**G**	21						**G**	1	w1	1-	362.0338	362.0329	2.49

Table S2: Peak list table on the fragments annotated on the sequence coverage map for Fomivirsen and that suffice to provide full sequence coverage, from the **UVPD MS²** spectrum on $[M - 9H]^{9-}$. Full FAST MS output of fragment ion peaks of Fomivrisen $[M - 9H]^{9-}$ is provided in supplementary Table S7 (separate Excel file).

nt	Position (5')	Peak assignment	Charge state (z)	Observed m/z	Theoretical m/z	Mass error (ppm)	nt	Position (3')	Peak assignment	Charge state (z)	Observed m/z	Theoretical m/z	Mass error (ppm)
**G**	1	c1	1-	344.0224	344.0224	0.00	**G**	21					
**C**	2	c2	1-	649.0467	649.0459	1.23	**C**	20	w20	7-	917.3509	917.3502	0.76
**G**	3	a3	1-	898.1318	898.1321	-0.33	**G**	19					
**T**	4	a4	2-	608.5738	608.574	-0.33	**T**	18					
**T**	5	a5	2-	768.5858	768.5856	0.26	**T**	17	w17	6-	908.7327	908.7303	2.64
**T**	6	c6	3-	650.7127	650.7102	3.84	**T**	16	w16	6-	855.3972	855.3931	4.79
**G**	7	a7•	3-	733.4042	733.403	1.64	**G**	15	y15	6-	786.0649	786.0654	-0.64
**C**	8	a8	3-	835.414	835.4134	0.72	**C**	14					
**T**	9						**T**	13	w13	6-	693.7155	693.7137	2.59
**C**	10	a10•	3-	1043.4345	1043.4264	7.76	**C**	12	w12	5-	768.6562	768.6533	3.77
**T**	11	a11	4-	862.5785	862.5757	3.25	**T**	11	w11	5-	707.6505	707.6486	2.68
**T**	12						**T**	10	w10	5-	643.6472	643.644	4.97
**C**	13	a13	5-	814.8706	814.8684	2.70	**C**	9	w9	4-	724.805	724.801	5.52
**T**	14	a14	5-	878.8751	878.8731	2.28	**T**	8	w8	4-	648.5481	648.5451	4.63
**T**	15	a15	6-	785.5573	785.5635	-7.89	**T**	7	w7	3-	758.3905	758.3882	3.03
**C**	16	a16	6-	836.409	836.4008	9.80	**C**	6	w6	3-	651.7151	651.7138	1.99
**T**	17	a17	6-	889.7408	889.738	3.15	**T**	5	y5	2-	777.5927	777.5908	2.44
**T**	18	a18•	7-	808.2105	808.2063	5.20	**T**	4	y4	2-	617.5807	617.5793	2.27
**G**	19	a19	7-	857.5001	857.4962	4.55	**G**	3	y3	1-	916.1433	916.1426	0.76
**C**	20						**C**	2	y2	1-	571.1135	571.113	0.88
**G**	21						**G**	1	w1	1-	362.0329	362.0329	0.00

Table S3: Peak list table on the fragments annotated on the sequence coverage map for Fomivirsen and that suffice to provide full sequence coverage, from the **EDD-IRMPD MS^3** spectrum on $[M-9H]^{9-\bullet}$. Full FAST MS output of fragment ion peaks of Fomivrisen $[M-9H]^{9-\bullet}$ is provided in supplementary Table S7 (separate Excel file).

nt	Position (5')	Peak assignment	Charge state (z)	Observed m/z	Theoretical m/z	Mass error (ppm)	nt	Position (3')	Peak assignment	Charge state (z)	Observed m/z	Theoretical m/z	Mass error (ppm)
**G**	1	c1•	1-	343.0169	343.0145	7.00	**G**	21					
**C**	2	a2•	1-	552.096	552.0946	2.54	**C**	20					
**G**	3	a3•	1-	897.1268	897.1242	2.90	**G**	19					
**T**	4	a4•	2-	608.0742	608.0701	6.74	**T**	18	w18	8-	721.2998	721.2988	1.39
**T**	5	a5	2-	768.5872	768.5856	2.08	**T**	17	w17•	7-	778.7744	778.7678	8.47
**T**	6	a6•	3-	618.3945	618.3931	2.26	**T**	16					
**G**	7	a7	3-	733.7406	733.7389	2.32	**G**	15					
**C**	8	a8•	4-	626.058	626.0563	2.72	**C**	14	w14•	6-	744.3881	744.383	6.85
**T**	9	a9•	4-	706.0647	706.0621	3.68	**T**	13	w13•	6-	693.549	693.5458	4.61
**C**	10	a10	4-	1043.4315	1043.4264	4.89	**C**	12	w12	5-	768.6566	768.6533	4.29
**T**	11	a11•	4-	862.3276	862.3237	4.52	**T**	11	w11	5-	707.6548	707.6486	8.76
**T**	12	a12•	5-	753.6672	753.6622	6.63	**T**	10	w10	5-	643.6498	643.644	9.01
**C**	13						**C**	9					
**T**	14	a14•	6-	732.0628	732.0584	6.01	**T**	8	w8	4-	648.5502	648.5451	7.86
**T**	15	a15•	6-	785.4004	785.3956	6.11	**T**	7	w7	4-	568.5434	568.5393	7.21
**C**	16	a16	7-	716.7726	716.7711	2.09	**C**	6	w6•	3-	651.3795	651.3779	2.46
**T**	17	a17•	7-	762.3469	762.3447	2.89	**T**	5	w5	2-	825.5655	825.5626	3.51
**T**	18	a18	7-	808.2106	808.2063	5.32	**T**	4	w4•	2-	665.0492	665.0471	3.16
**G**	19						**G**	3	w3•	2-	505.0374	505.0355	3.76
**C**	20						**C**	2	w2•	1-	666.054	666.0486	8.11
**G**	21						**G**	1	w1•	1-	361.0221	361.0251	-8.31

Table S4: Peak list table on the fragments annotated on the sequence coverage map for Fomivirsen and that suffice to provide full sequence coverage, from the **EDD-$_{\mathbf{R}}$CID MS^{3}** spectrum on [M − 9H]$^{9-\bullet}$. Full FAST MS output of fragment ion peaks of Fomivrisen [M − 9H]$^{9-\bullet}$ is provided in supplementary Table S7 (separate Excel file).

**nt**	**Position (5')**	**Peak assignment**	**Charge state (z)**	**Observed m/z**	**Theoretical m/z**	**Mass error (ppm)**	**nt**	**Position (3')**	**Peak assignment**	**Charge state (z)**	**Observed m/z**	**Theoretical m/z**	**Mass error (ppm)**
**G**	1	c1$^{\bullet}$	1-	343.0154	343.0145	2.62	**G**	21					
**C**	2	a2$^{\bullet}$	1-	552.096	552.0946	2.54	**C**	20	w20	8-	802.561	802.5555	6.85
**G**	3	a3$^{\bullet}$	2-	448.0614	448.0585	6.47	**G**	19	w19$^{\bullet}$	8-	764.3053	764.3016	4.84
**T**	4	a4$^{\bullet}$	2-	608.0742	608.0699	7.07	**T**	18	w18	8-	721.3019	721.2988	4.30
**T**	5	a5$^{\bullet}$	2-	768.081	768.0817	-0.91	**T**	17	w17$^{\bullet}$	7-	778.7738	778.7678	7.70
**T**	6	a6$^{\bullet}$	3-	618.3945	618.3931	2.26	**T**	16	w16$^{\bullet}$	7-	733.0556	733.0502	7.37
**G**	7	a7$^{\bullet}$	3-	733.407	733.403	5.45	**G**	15	w15$^{\bullet}$	7-	687.188	687.1886	-0.87
**C**	8	a8$^{\bullet}$	4-	626.0589	626.0563	4.15	**C**	14	w14$^{\bullet}$	5-	893.4615	893.4611	0.45
**T**	9	a9$^{\bullet}$	4-	706.0645	706.0621	3.40	**T**	13	w13$^{\bullet}$	6-	693.549	693.5458	4.61
**C**	10	a10$^{\bullet}$	3-	1043.4334	1043.4264	6.71	**C**	12	w12$^{\bullet}$	5-	768.4589	768.4517	9.37
**T**	11	a11$^{\bullet}$	4-	862.3253	862.3237	1.86	**T**	11	w11$^{\bullet}$	5-	707.4423	707.447	-6.64
**T**	12	a12$^{\bullet}$	5-	753.6673	753.6622	6.77	**T**	10	w10$^{\bullet}$	5-	643.4409	643.4424	-2.33
**C**	13	a13	5-	814.8735	814.8684	6.26	**C**	9	w9	4-	724.808	724.801	9.66
**T**	14	a14$^{\bullet}$	6-	732.0603	732.0584	2.60	**T**	8	w8	4-	648.5481	648.5451	4.63
**T**	15	a15$^{\bullet}$	6-	785.3941	785.3956	-1.91	**T**	7	w7	4-	568.5422	568.5393	5.10
**C**	16	a16$^{\bullet}$	6-	836.2386	836.2328	6.94	**C**	6	w6$^{\bullet}$	3-	651.3804	651.3779	3.84
**T**	17	a17$^{\bullet}$	7-	762.3475	762.3447	3.67	**T**	5	w5	2-	825.5644	825.5626	2.18
**T**	18	a18$^{\bullet}$	7-	808.21	808.2063	4.58	**T**	4	w4$^{\bullet}$	2-	665.052	665.0471	7.37
**G**	19	a19$^{\bullet}$	8-	750.0509	750.0573	-8.53	**G**	3	w3	2-	505.036	505.0355	0.99
**C**	20	a20$^{\bullet}$	8-	788.1895	788.1852	5.46	**C**	2	w2$^{\bullet}$	1-	666.0531	666.0486	6.76
**G**	21						**G**	1	w1$^{\bullet}$	1-	361.0221	361.0251	-8.31

Table S5: Peak list table on the fragments annotated on the sequence coverage map for Fomivirsen and that suffice to provide full sequence coverage, from the **$_R$CID MS^2** spectrum on $[M-9H]^{9-}$. Full FAST MS output of fragment ion peaks of Fomivrisen $[M-9H]^{9}$ is provided in supplementary Table S7 (separate Excel file).

**nt**	**Position (5')**	**Peak assignment**	**Charge state (z)**	**Observed m/z**	**Theoretical m/z**	**Mass error (ppm)**	**nt**	**Position (3')**	**Peak assignment**	**Charge state (z)**	**Observed m/z**	**Theoretical m/z**	**Mass error (ppm)**
**G**	1-	c1	1-	344.0228	344.0224	0.12	**G**	21					
**C**	2	a2-C	1-	442.0598	442.0591	0.16	**C**	20	y20	8-	790.5602	790.5625	-0.29
**G**	3	a3-G	1-	747.085	747.0827	0.31	**G**	19	y19	8-	752.5641	752.5599	0.56
**T**	4	c4	2-	656.5482	656.5457	0.38	**T**	18	y18	7-	810.7843	810.7792	0.63
**T**	5	b5	2-	777.5939	777.5908	0.40	**T**	17	y17	7-	765.0583	765.0616	-0.43
**T**	6	c6	3-	650.7138	650.7102	0.55	**T**	16	y16	6-	839.4066	839.4026	0.48
**G**	7	a7-G	2-	1025.5921	1025.5873	0.47	**G**	15	y15	6-	786.0696	786.0654	0.53
**C**	8	a8-C	3-	798.4022	798.399	0.40	**C**	14	w14	6-	744.5568	744.551	0.78
**T**	9	b9	4-	710.8206	710.8167	0.55	**T**	13	w13	6-	693.7173	693.7137	0.52
**C**	10	a10-C	4-	754.8124	754.8091	0.44	**C**	12	y12	5-	749.4681	749.4646	0.47
**T**	11	a11	4-	862.5798	862.5757	0.48	**T**	11	w11	5-	707.6526	707.6486	0.57
**T**	12	b12	5-	757.4699	757.4658	0.54	**T**	10	y10	4-	780.8248	780.8209	0.50
**C**	13	a13-C	5-	792.6643	792.6598	0.57	**C**	9	y9	4-	700.8184	700.8151	0.47
**T**	14	a14	5-	878.8772	878.8731	0.47	**T**	8	y8	3-	833.0823	833.0814	0.11
**T**	15	b15	6-	788.5686	788.5653	0.42	**T**	7	w7	4-	568.5428	568.5393	0.62
**C**	16	a16	6-	836.4138	836.4008	1.55	**C**	6	w6	4-	488.5367	488.5335	0.66
**T**	17	a17	6-	889.7431	889.738	0.57	**T**	5	w5	3-	550.0422	550.0393	-0.53
**T**	18	c18	7-	821.9151	821.9125	0.32	**T**	4	y4	2-	617.5822	617.5793	0.47
**G**	19	c19	8-	762.1805	762.1762	0.56	**G**	3	y3	2-	457.5693	457.5677	0.35
**C**	20	a20-C	8-	774.4526	774.4308	2.81	**C**	2	w2	1-	667.0582	667.0564	0.27
**G**	21						**G**	1	w1	1-	362.0337	362.0329	0.22

Table S6: Peak list table on the fragments annotated on the sequence coverage map for Fomivirsen and that suffice to provide full sequence coverage, from the **IRMPD MS²** spectrum on $[M - 9H]^{9-}$. Full FAST MS output of fragment ion peaks of Fomivrisen $[M - 9H]^{9-}$ is provided in supplementary Table S7 (separate Excel file).

nt	Position (5')	Peak assignment	Charge state (z)	Observed m/z	Theoretical m/z	Mass error (ppm)	nt	Position (3')	Peak assignment	Charge state (z)	Observed m/z	Theoretical m/z	Mass error (ppm)
**G**	1	c1	1-	344.0233	344.0224	0.26	**G**	21					
**C**	2	a2-C	1-	442.0599	442.0591	0.18	**C**	20					
**G**	3	a3-G	1-	747.0845	747.0827	0.24	**G**	19	y19	8-	752.4386	752.4346	0.53
**T**	4	c4	2-	656.5488	656.5457	0.47	**T**	18	y18	7-	810.7857	810.7792	0.80
**T**	5	b5	2-	777.594	777.5908	0.41	**T**	17	y17	7-	765.0577	765.0616	-0.51
**T**	6	b6	3-	650.7132	650.7102	0.46	**T**	16	y16	6-	839.395	839.4026	-0.91
**G**	7	a7-G	2-	1025.5919	1025.5873	0.45	**G**	15	y15	6-	786.0679	786.0654	0.32
**C**	8	a8-C	3-	798.4024	798.399	0.43	**C**	14	w14	7-	638.0448	638.0427	0.33
**T**	9	b9	4-	710.8207	710.8167	0.56	**T**	13	y13	5-	813.4731	813.4693	0.47
**C**	10	a10-C	4-	754.8115	754.8091	0.32	**C**	12	x12	5-	765.0577	765.0512	0.85
**T**	11	a11	4-	862.5809	862.5757	0.60	**T**	11	w11	5-	707.6523	707.6486	0.52
**T**	12	b12	5-	757.4689	757.4658	0.41	**T**	10	y10	4-	780.8244	780.8209	0.45
**C**	13	a13-C	5-	792.6635	792.6598	0.47	**C**	9	y9	4-	700.8193	700.8151	0.60
**T**	14						**T**	8	y8	3-	833.0214	833.0814	-7.20
**T**	15	b15	6-	788.5617	788.5653	-0.46	**T**	7	y7	4-	568.5426	568.5393	0.58
**C**	16	a16-C	6-	817.8979	817.8936	0.53	**C**	6	w6	4-	488.5368	488.5335	0.68
**T**	17						**T**	5	w5	3-	550.0422	550.0393	0.53
**T**	18						**T**	4	w4	3-	443.3664	443.3649	0.34
**G**	19	c19	8-	762.1806	762.1762	0.58	**G**	3	y3	1-	916.1448	916.1419	0.32
**C**	20	a20-C	8-	774.4352	774.4308	0.57	**C**	2	w2	1-	667.0582	667.0564	0.27
**G**	21						**G**	1	w1	1-	362.0335	362.0329	0.17

Table S7 (see .XLSX file): Full fragment lists for Fomivirsen 9– (all fragmentation methods).

Table S8: Summary of total number of peaks (complete isotopic distributions) and product ions observed by six fragmentation methods for Fomivirsen (charge state 10–, closed shell in MS² and open shell in MS³).

	**MS²**				**MS³**	
	$_{R}$**CID**	**IRMPD**	**UVPD**	**EDD**	**EDD-IRMPD**	**EDD-**$_{R}$**CID**
Total number of peaks	630	633	744	745	440	356
Number of assigned peaks	199	201	204	210	178	138
% assigned peaks	31.6	31.8	27.4	28.2	40.5	38.8
Among assigned peaks:						
% 5' fragments (including *a*-B)	39.2	32.3	29.4	31.4	32.0	29.7
% 3' fragments	29.1	29.4	27.9	29.5	30.3	27.5
% other fragments + base losses	31.7	37.8	25.5	1.9	12.4	7.2
% radical fragments	0	0	17.2	37.1	25.3	35.5
Predominant product ion types	*a-B,a, y*	*a-B, w, y*	*a,w*	*a, w*	*a, a•, w,w•*	*a, a•, w, w•*
Sequence coverage	100%	100%	100%	100%	100%	100%

Table S9: Summary of total number of peaks (complete isotopic distributions) and product ions observed by six fragmentation methods for Fomivirsen (charge state 5–, closed shell in MS² and open shell in MS³).

	**MS²**				**MS³**	
	$_{R}$**CID**	**IRMPD**	**UVPD**	**EDD**	**EDD-IRMPD**	**EDD-**$_{R}$**CID**
Total number of peaks	333	381	419	185	625	509
Number of assigned peaks	115	126	105	13	334	189
% assigned peaks	34.5	33.1	25.1	7.0	53.4	37.1
Among assigned peaks:						
% 5' fragments (including *a*-B)	27.0	21.4	17.1	15.4	22.8	27.0
% 3' fragments	26.1	18.3	15.2	23.1	15.3	20.1
% other fragments + base losses	47.0	60.3	45.7	30.8	28.1	13.2
% radical fragments	0	0	21.9	30.8	33.8	39.7
Predominant product ion types	*a-B, w, w-B*	*a-B, w, w-B*	*w, w•, w-B*	*w, w•*	*a,a-B, a•, w,w-B, w•*	*a, a•, w, w•*
Sequence coverage	95%	85%	62%	23%	100%	100%

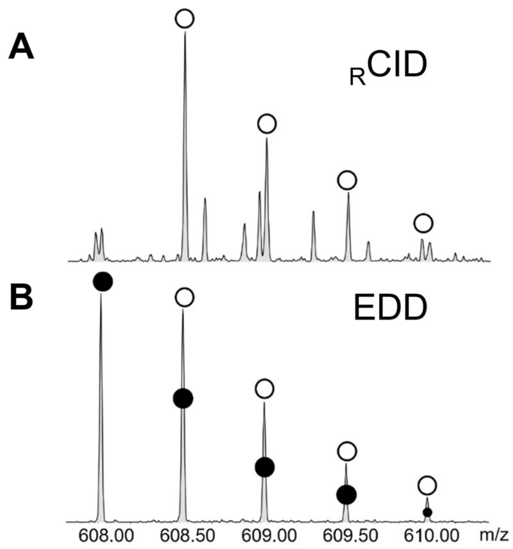

Figure S4: Isotopic distribution of the a_4^{2-} fragment ion of fomivirsen, $[M-9H]^{9-}$ electrosprayed from 1 mM aqueous NH_4OAc A) $_R$CID showing closed-shell isotopic distribution and B) EDD showing a mixture of open and closed-shell isotopic distribution. Black circles indicate radical ions, while open circles indicate closed-shell ions. The two species differ by one hydrogen mass divided by the charge state (1.008 Da/z).

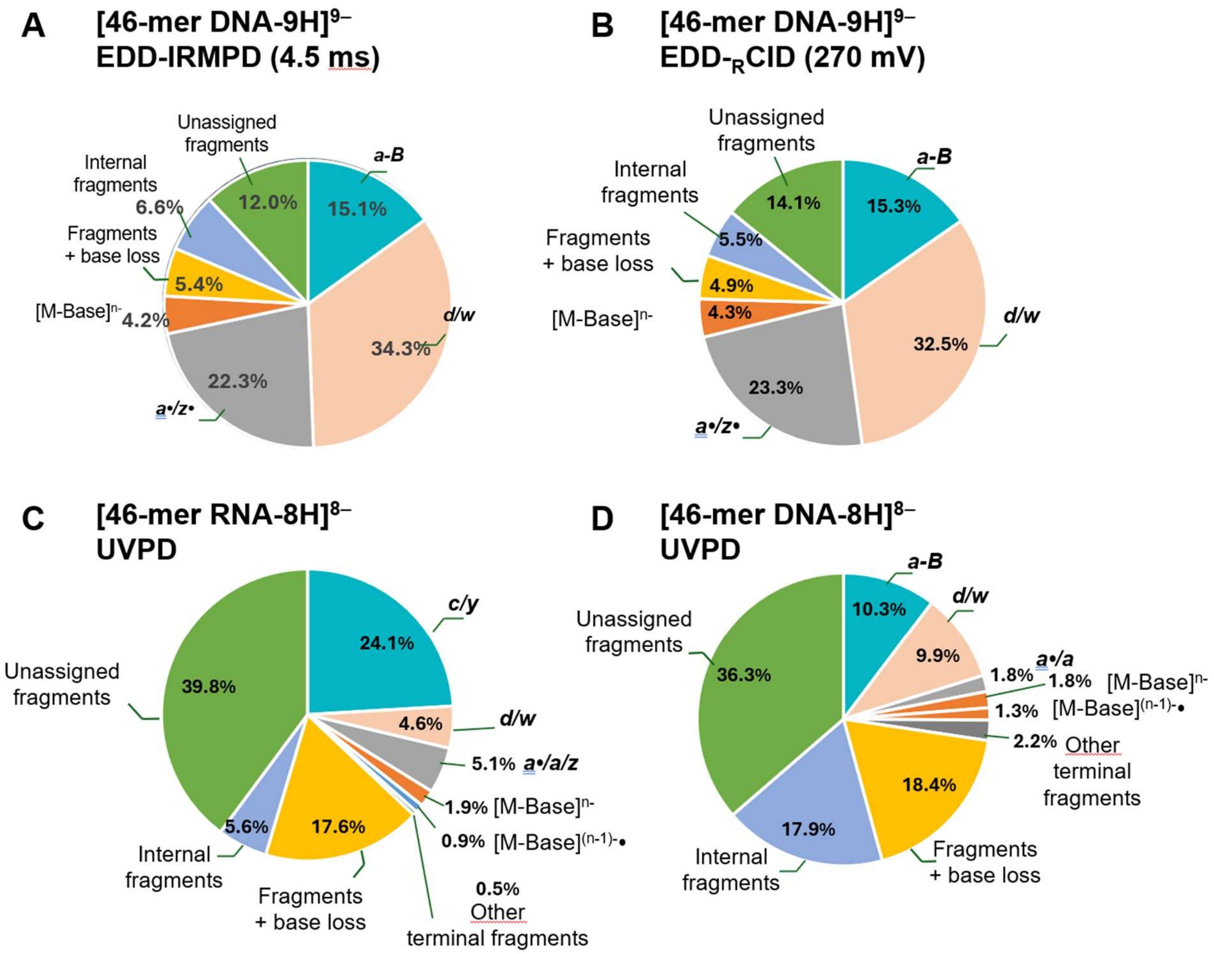

Figures S5: (A-B) Comparison of the fraction of peaks assigned to each fragment category for EDD performed on $[M-9H]^{9-}$ DNA, followed by IRMPD or $_R$CID on re-isolated $[M-8H]^{8-\bullet}$. All fragment assignments are provided in supporting information Table S8 (separate Excel file). Activation energies: EDD was performed using 30 eV electron kinetic energy and 50 ms irradiation time. Subsequent vibrational activation was achieved using 4.5 ms IRMPD (9.3 µm, 50% power) or 270 mV for $_R$CID. **(C-D)** Comparison of 46-mer RNA (A) and DNA (B) sequence coverage with UVPD alone on $[M-8H]^{8-}$. UVPD parameters consisted of one pulse at 0.6 mJ per pulse. All fragment assignments are provided in supporting information Table S8 (separate Excel file).

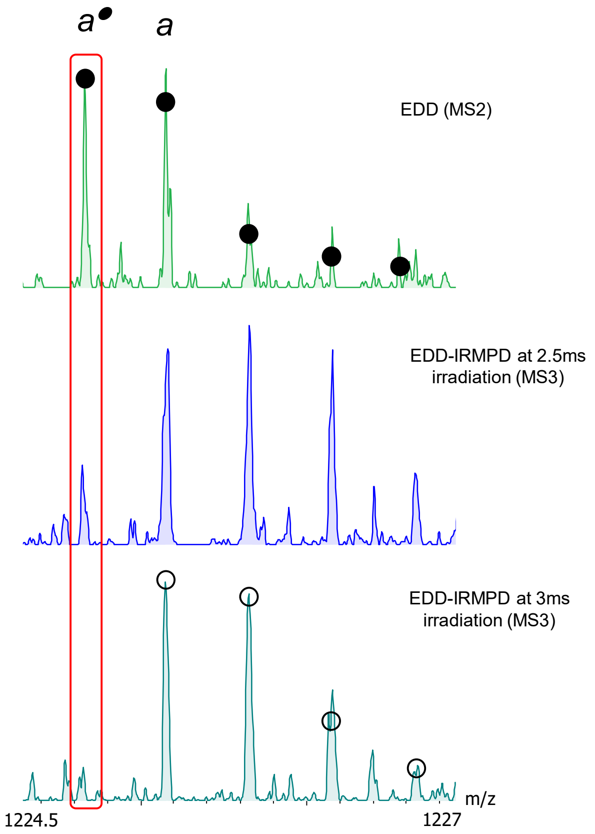

Figures S6: Effect of IRMPD irradiation time on the abundance of *a*• radical fragment generated by EDD of the 46mer RNA $[M\text{-}8H]^{9-}$. Black and white circles correspond to theoretical isotopic distributions for radical and closed-shell *a* fragment respectively. With EDD alone (MS^2), we almost have full radical $a^{\bullet}$ ions. Upon activation with IRMPD at 2.5 irradiation, we have a mixture of radical and closed-shell *a* fragments. At higher energies (3 ms IRMPD), the isotopic distribution corresponds to a full-closed-shell ion.

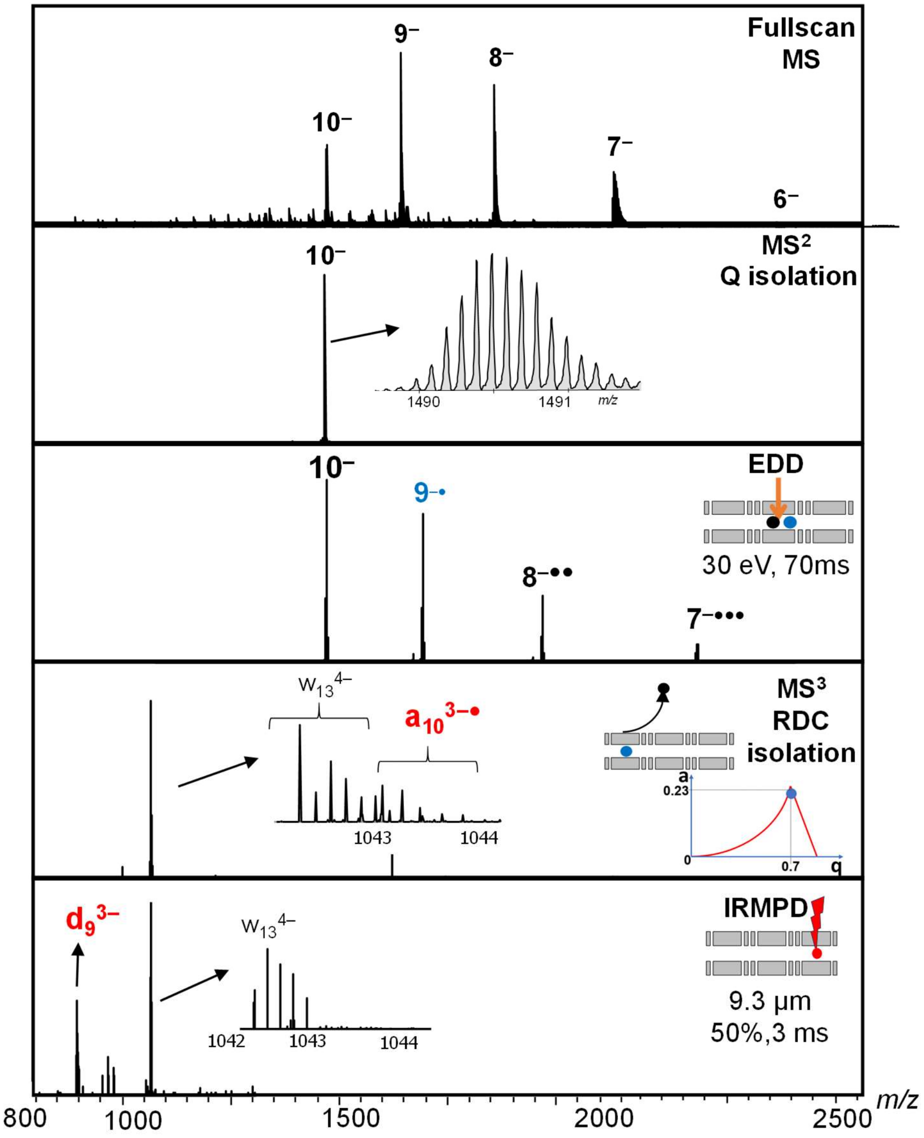

Figures S7: MS^3 dissociation of radical fragment into a closed-shell ion. From the fullscan MS spectrum of the 46-mer RNA UCUAAGUAAAAUUGGGUGGGUGGGUGGGUUUGUCGGUAUCUCGGUU, the charge state 10- (m/z 1490.9) is isolated using the quadrupole. The ions are accumulated in section Q5 for 250 ms and submitted to EDD. 70 ms of irradiation with electron of kinetic energy 30 eV is used to produce radicals ($9^{-\bullet}$, $8^{-\bullet\bullet}$, $7^{-\bullet\bullet\bullet}$). The radical $a_{10}^{3-\bullet}$ (m/z 1043.15) is isolated from the EDD spectrum in a MS^3 stage using resolving DC by changing the drive frequency of the Omnitrap. It was actually co-isolated with a *w* ion, but upon irradiation for 3 ms (IRMPD, 9.3 µM, 50% power), $a_{10}^{3-\bullet}$ disappears and the first resulting fragment is a d_9^{3-}.

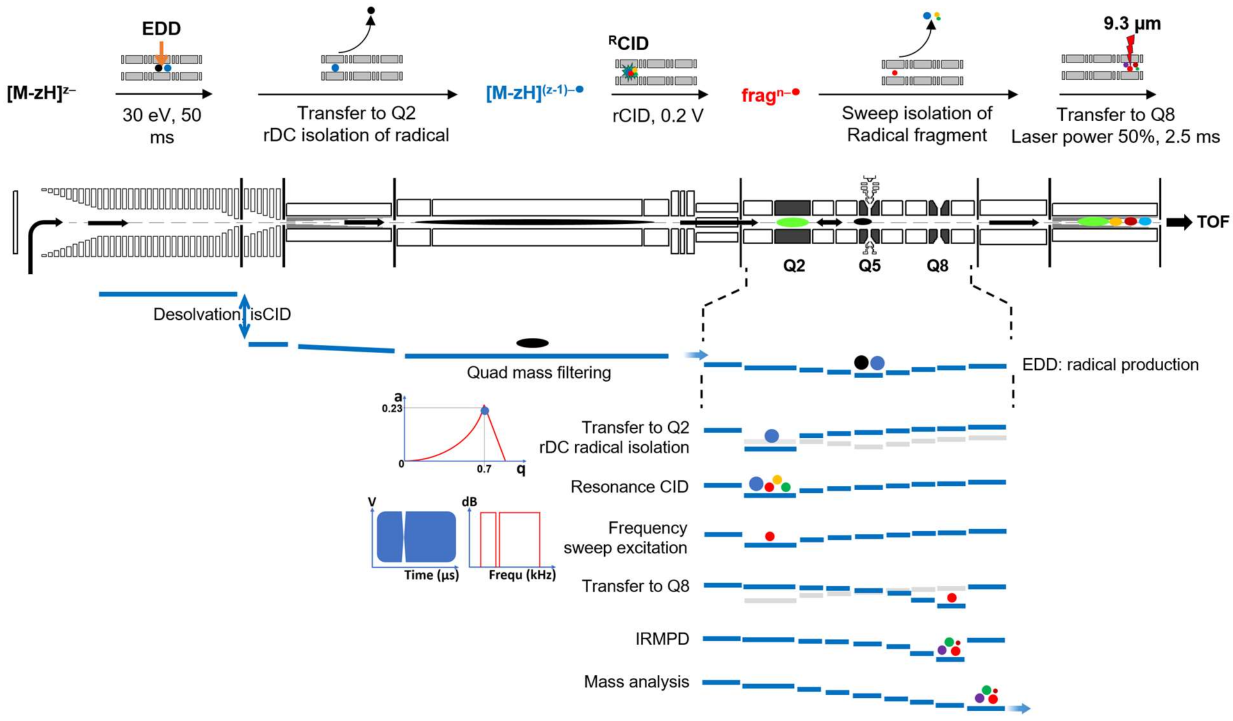

Figures S8: Schematic representation of the **MS⁴** experiment reported in main text Figure 7 and supplementary Figure S10. **MS²**: the precursor ion is selected using the quadrupole and accumulated in Q5. The ion undergoes EDD for 50 ms and the resulting ions are transferred to Q2. **MS³**: in Q2, the radical ion resulting from a single electron loss is isolated using resolving DC and submitted to resonance CID, producing both closed shell and radical fragments. **MS⁴**: One of the radical fragment is re-isolated using a frequency sweep excitation with a notch corresponding to its secular frequency and then submitted to resonance CID. The fragment ions are transferred toward the mass analyzer. The complete sequence takes 350 ms when using an accumulation time of 80 ms before the EDD process. The blue lines schematically represent the DC voltages applied to the different sections of the Omnitrap. The light gray lines represent the voltages from the previous step for ease of visualization.

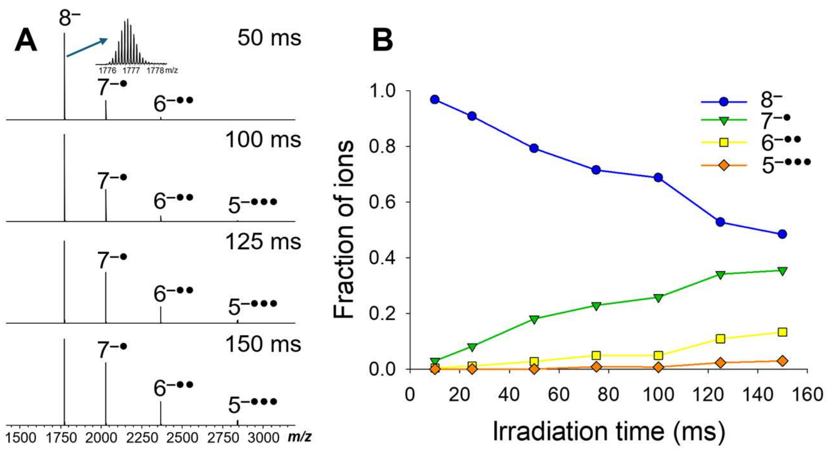

Figure S9: Influence of the EDD irradiation time on the 46-mer DNA $[M-8H]^{8-}$: A) EDD MS^2 spectra, and B) extracted peak areas. The kinetic energy of the electron is 30 eV.

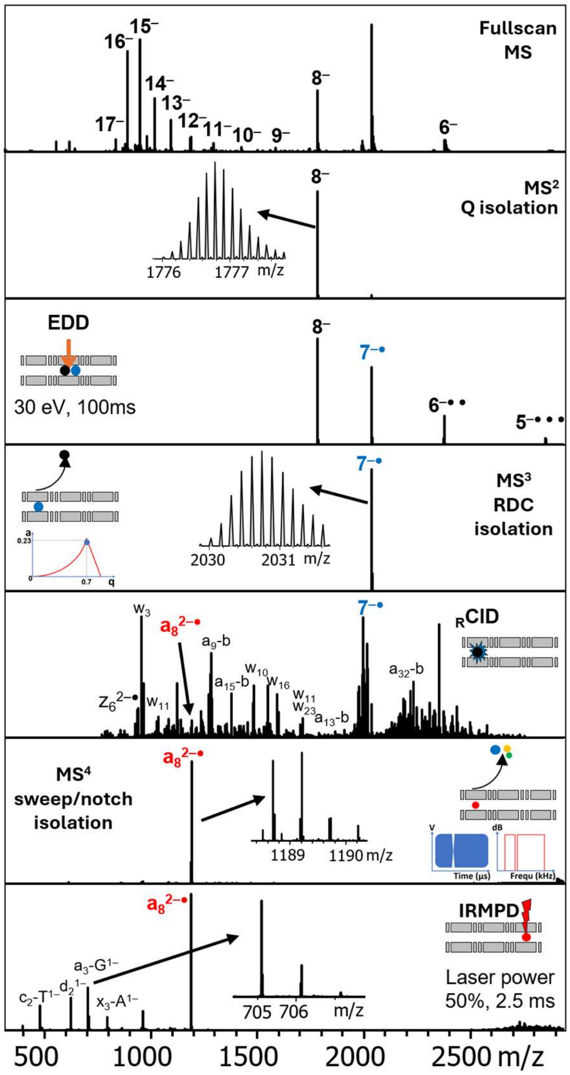

Figure S10: MS^4 to elucidate the fate of an *a*• radical fragment from the 46-mer DNA. From the full scan MS spectrum, the charge state 8– (m/z 1776.9) is isolated using the quadrupole. A description Q1—Q9 DC gradient programming for each step is provided in Supporting Information Figure S8. The ions are accumulated in Q5 for 100 ms and submitted to EDD. 150 ms of irradiation with electron of kinetic energy 30 eV is used to produce radicals (the dependence of radical product ion yields on the irradiation time is shown in Supporting Information Figure S9). The radical $7^{-\bullet}$ (m/z 2030.7) is isolated in a MS^3 stage using resolving DC by changing the drive frequency of the Omnitrap. The ions then undergo resonance CID during 10 ms (0.2 V). For the MS^4 stage, the radical fragment $a_8^{2-\bullet}$, which is a very minor product in MS^3, is re-isolated using a sweep of frequencies including a notch corresponding to its secular frequency. After transfer to section Q8, the radical fragment is irradiated for 2.5 ms (IRMPD, 9.3 µm, 50% power). The resulting fragments are a variety of closed shell ions. The total duration of the sequence is 350 ms, and the reported MS^4 spectrum was recorded in 3 minutes.
